# THE EFFECTIVENESS OF INTERNET-BASED INTERVENTIONS IN REDUCING STRESS EXPERIENCED BY OLDER INFORMAL CAREGIVERS

**DOI:** 10.1093/geroni/igac059.2227

**Published:** 2022-12-20

**Authors:** Abdulmajeed Altukhys, Richard Beaulaurier

**Affiliations:** Florida International University, Miami, Florida, United States; Florida International University, Miami, Florida, United States

## Abstract

**Purpose:**

This systematic review assesses and provides a critical review of the effectiveness of internet-based interventions to decrease informal caregiver stress. Method: A systematic review of the following databases: PsycINFO, Social Service Abstract, Social Work Abstract, PubMed, and Cochrane Library was conducted using terms related to informal caregivers and internet-based interventions from January 2010-February 2022. Only data conducting interventions in the U.S. included. This systematic review reviewed the quality of articles on the methods of randomized and non-randomized trials using the approaches recommended by the Cochrane Handbook for systematic review for intervention. The review was reported using a PRISMA chart.

**Results:**

Eight studies met the review criteria. Most of the studies showed positive benefits in reducing caregiver stress. There were no clear patterns as to the variables such as study duration and complexity of intervention associated with better outcomes, although earlier studies typically had more negative outcomes.

**Conclusions:**

Internet-based interventions can develop solutions to decrease the physical and psychological consequences resulting from caregiving and can help empower older caregivers of people with chronic diseases. Internet-based interventions were mainly effective in reducing aspects of caregiver stress and improving their well-being. Further studies can assess outcomes for older informal caregivers and their recipients’ health, different technology delivery methods, and the cost of such interventions are needed to examine the psychological impacts of COVID-19 on older informal caregivers.

